# CD148 Serves as a Prognostic Marker of Gastric Cancer and Hinders Tumor Progression by Dephosphorylating EGFR

**DOI:** 10.7150/jca.40955

**Published:** 2020-02-19

**Authors:** Yiting Sun, Song Li, Wenbin Yu, Cheng Chen, Teng Liu, Lanbo Li, Di Zhang, Zeyi Zhao, Jing Gao, Xiao Wang, Duanbo Shi, Lian Liu

**Affiliations:** 1 Department of Medical Oncology, Cancer Center, Qilu Hospital of Shandong University, Jinan, Shandong, 250012, China;; 2 Department of Medical Oncology, National Cancer Center/National Clinical Research Center for Cancer/Cancer Hospital, Chinese Academy of Medical Sciences & Peking Union Medical College, Beijing, 100021, China;; 3 Department of General Surgery, Qilu Hospital of Shandong University, Jinan, Shandong, 250012, China;; 4 Animal Laboratory, Qilu Hospital of Shandong University, Jinan, Shandong, 250012, China;; 5 Department of Pathology, School of Medicine, Shandong University, Jinan, Shandong, 250012, China;; 6 Department of Pathology, Qilu Hospital of Shandong University, Jinan, Shandong, 250012, China.

**Keywords:** gastric cancer, CD148, epidermal growth factor receptor, protein tyrosine phosphatase

## Abstract

CD148 is a member of the receptor-type protein tyrosine phosphatase family encoded by the *PTPRJ* gene and has controversial impacts on cancers. In this study, we investigated the clinical significance of CD148 in gastric cancer and the possible mechanisms. Suppressed CD148 expression indicated adverse pathological features and poor outcomes in gastric cancer patients. CD148 overexpression impeded tumor proliferation, motility, and invasiveness, while CD148 knock-down or knockout promoted the ability of gastric cancer cells to grow and metastasize *in vitro* and *in vivo*. Mechanistically, CD148 negatively regulated EGFR phosphorylation of multiple tyrosine residues, including Y1173, Y1068, and Y1092, and remarkably inhibited downstream PI3K/AKT and MEK/ERK pathways. *In silico* analysis revealed that gene deletions or missense/truncated mutations of *PTPRJ* gene rarely occurred in gastric cancers. Instead, a 3' UTR-specific methylation might regulate CD148 expression, and the potential regulators were TET2 and TET3. Collectively, our results suggest that CD148 is a convincing prognostic marker as well as a potential therapeutic target for gastric cancer.

## Introduction

Gastric cancer is the fourth most common malignancy and the second-leading cause of cancer-related deaths worldwide 1. Lacking specific symptoms, early detection of gastric cancer remains challenging, and diagnosis is frequently made when tumors become unresectable 2. Meanwhile, gastric cancer has high recurrence and distant metastasis rates and responds poorly to traditional radiotherapy and chemotherapy 2. Therefore, gastric cancer patients, especially those with distant metastasis, have a poor overall prognosis 3, 4. The median survival time of distant metastasis is less than 12 months. Compared to conventional strategies, such as chemotherapy and radiotherapy, targeted therapies that have favorable effects on other malignancies show limited overall benefit for patients with advanced gastric cancer 5-8. In addition, anti-PD-1 immunotherapy is only recommended as the third-line treatment and has a limited effect on the survival of patients with advanced gastric cancer 9, 10. Therefore, understanding the molecular mechanisms underlying carcinogenesis and progression is critical for identifying early detection markers and candidate intervention targets to improve overall survival and quality of life in patients with gastric cancer.

Sequential phosphorylation is one of the common mechanisms regulating the biological behavior of malignant tumors. This process is regulated by two types of enzymes with reverse effects, the protein tyrosine kinases (PTKs) and the protein tyrosine phosphatases (PTPs). By dephosphorylating target proteins, PTPs counter the activities of PTKs that are involved in signaling transductions and influence the malignant biological processes of tumors 11. The receptor-type PTPs (PTPRs) are a subgroup of PTPs that share a transmembrane domain and distribute along cell membranes 11. CD148, also named density-enhanced phosphatase 1 (DEP-1) or PTP receptor type J (PTPRJ), is a member of the PTPR family encoded by the *PTPRJ* gene 12. CD148 may regulate signal transduction by dephosphorylation of multiple key proteins in tumors, such as ERK1/2 13, VEGFR 14, Src 15, and thus affect the corresponding biological consequences in multiple aspects. Moreover, CD148 suppresses phosphorylating activity and accelerates the degradation of EGFR, consequently blocking downstream signaling 16. However, the effects of CD148 on EGFR in certain cancers have not yet been reported.

Previous studies have reported multiple roles of CD148 in cancers. For example, CD148 plays tumor-suppressing roles in colon 17, breast 18, and thyroid cancers 19. Polymorphisms of the *PTPRJ* gene also affect susceptibility to lung, head and neck, colorectal, and esophageal cancers 20. In contrast, CD148 may also promote metastasis in certain cancers, including breast cancer 21 and glioma cells 22. Despite these roles of CD148 in cancers, the biological significance of CD148 in gastric cancer remains unclear.

In the present study, we investigated CD148 expression and its relationship with clinicopathological features and prognosis of patients with gastric cancer, as well as the possible expression-regulating mechanisms. Furthermore, *in vitro* and *in vivo* experiments demonstrated that CD148 inhibited the malignancy of gastric cancer cells. These results reveal that CD148 plays a suppressing role in the development and progression of gastric cancer and has a prognostic value in gastric cancer.

## Materials and Methods

### Cell culture

The BGC-823, SGC-7901, and MKN45 cell lines were purchased from the Cell Bank, Chinese Academy of Science (Shanghai, China), and incubated at 37°C with 90% relative humidity and 5% CO_2_). Mycoplasma testing has been done for the cell lines, and no contamination was detected. Cells were passaged when confluence reached 80 to 90%. Exponentially growing cells were used for all experiments.

### Transfection

The CD148 overexpression plasmid was made by inserting CD148 mRNA into an expressing vector, and then was used for forced expression in cells, with the empty vector as a negative control. The siRNA targeting CD148 (Santa Cruz, Dallas, Texas, USA) was used for CD148-knockdown, with a scrambled siRNA (Santa Cruz, Dallas, Texas, USA) as the negative control. Lipofectamine 2000 (Thermo Fisher Scientific, Waltham, MA, USA) was used for plasmid or siRNA transfection according to the manufacturer's instructions.

### Western blotting

Total proteins were extracted from cells or ultrasound-treated tumors by RIPA lysis buffer, phenylmethanesulfonylfluoride, and phosphatase inhibitor (all from Beyotime, Shanghai, China). Approximately 30 μg of protein of each sample were separated using SDS-PAGE, blotted to polyvinylidene difluoride membranes, and probed by antibodies. Anti-CD148, anti-EGFR, anti-EGFR (phosphor-Y1173), anti-EGFR (phosphor-Y1068), anti-EGFR (phosphor-Y1092), and anti-GAPDH antibodies were all purchased from Abcam (Cambridge, UK). The secondary anti-mouse and anti-rabbit antibodies were purchased from Affinity Biosciences (OH, USA). Protein bands were visualized by chemiluminescence kits (WBKLS0100, Millipore, Darmstadt, Germany) under MiniBIS Pro gel imaging system (DNR Bio Imaging Systems, Jerusalem, Israel).

### Proliferation assay and colony-formation assay

For the proliferation assay, 2 × 10^4^ cells were seeded into each well of 6-well plates. Cells were counted every 24 hours from days 0 to 5. Each point was replicated three times independently. For colony formation assays, six-well plates were seeded with 1000 suspended single cells. After incubation for 2 weeks, cell colonies were fixed with methanol, stained with crystal violet (Beyotime, Shanghai, China), and counted. Each well or plate was replicated three times independently.

### Migration and invasion assays

A Transwell system (8 µm pore size; Corning Incorporated, Corning, NY, USA) was used for invasion and migration assays according to the manufacturer's instructions. In the upper chambers, we added serum-free RPMI-1640 (Thermo Fisher Scientific) containing 1 × 10^5^ (for migration assays) and 2 × 10^5^ cells (for invasion assays). In addition, Matrigel (BD Biosciences, San Jose, CA, USA) was pre-coated in the upper chamber for the invasion assay. In the lower chambers, we added RPMI-1640 medium with 20% FBS (Sciencell, San Diego, CA, USA). After 48 hours, migrated and invaded cells were fixed with methanol, stained with crystal violet (Beyotime, Shanghai, China), and photographed under microscopy (TH4-200, Olympus, Tokyo, Japan).

### Immunohistochemistry staining

This study was approved by the Medical Ethical Committee of Qilu Hospital of Shandong University. Paraffin-embedded tissues were deposited in the Department of Pathology, Qilu Hospital of Shandong University. Sections were cut, baked at 60°C, deparaffinized with xylenes, and rehydrated. After antigen retrieval by EDTA (ZLI-9066, Zsbio, Beijing, China) and blocking by goat serum (SP-9001-2, Zsbio, Beijing, China), sections were stained with anti-CD148 antibody (Abcam, Cambridge, UK) using an immunohistochemistry staining kit (SP-9001, Zsbio, Beijing, China). Cell nuclei were stained with hematoxylin. Staining results were independently evaluated by three researchers, including two pathologists. The CD148 staining intensity was scaled as negative (-), weak (+), moderate (++), and strong staining (+++).

For animal studies, tumor xenografts were excised and fixed for immunohistochemistry analysis with a similar procedure as above. Anti-EGFR (phospho-Y1173) and the anti-Ki67 antibodies were purchased from Abcam (Cambridge, UK).

### CRISPR/Cas9 knockout

BGC 823 cells were co-transfected with CD148 CRISPR/Cas9 KO plasmid or control CRISPR/Cas9 knockout plasmid and HDR plasmid according to the manufacturer's protocol. Transfected cells were consequently selected by puromycin for two weeks. All plasmids and reagents were purchased from Santa Cruz (Dallas, Texas, USA).

### Animal studies

BGC823 cells with CD148 CRISPR/Cas9-knockout or control were subcutaneously injected into the left flank of 4-week-old BALB/c nude mice purchased from Beijing Vital River Laboratory Animal Technology Co., Ltd (Beijing, China). Six animals were used for each group and housed in pathogen-free facilities. Mice were euthanized 4 weeks post-injection. Tumor sizes and diameters were measured, and then tumors were excised and fixed for subsequent immunohistochemistry analysis. The animal study was approved by the Animal Ethical Committee of Qilu Hospital of Shandong University.

### *In silico* studies

The survival data from TCGA databases were collected using the online tool OncoLnc (http://www.oncolnc.org). Survival data from GEO databases were collected using the online tool Kaplan-Meier Plotter (http://kmplot.com) 23. Genomic alternation data were extracted from cBioPortal (http://www.cbioportal.org) 24, 25. Data of methylation levels around the *PTPRJ* gene were obtained from Wanderer (http://maplab.imppc.org/wanderer) 26. All parameters were set to default. RNA levels of CD148, DNMT1, DNMT3A, DNMT3B, DNMT3L, TET1, TET2, TET3, and TDG were extracted from TCGA databases (https://portal.gdc.cancer.gov).

### Statistical analyses

GraphPad Prism 7 was used for graphs and statistics. Unpaired *t*-tests were used to evaluate statistical significances of the mean values. Chi-square or Fisher's exact tests were used to compare ratios. Cox regression and Log-rank tests were used to compare survival. Pearson correlation tests were used to calculate correlations. A *P* value < 0.05 was considered statistically significant.

## Results

### Suppressed CD148 expression associates with poor prognosis in gastric cancer

**Figure 1:**
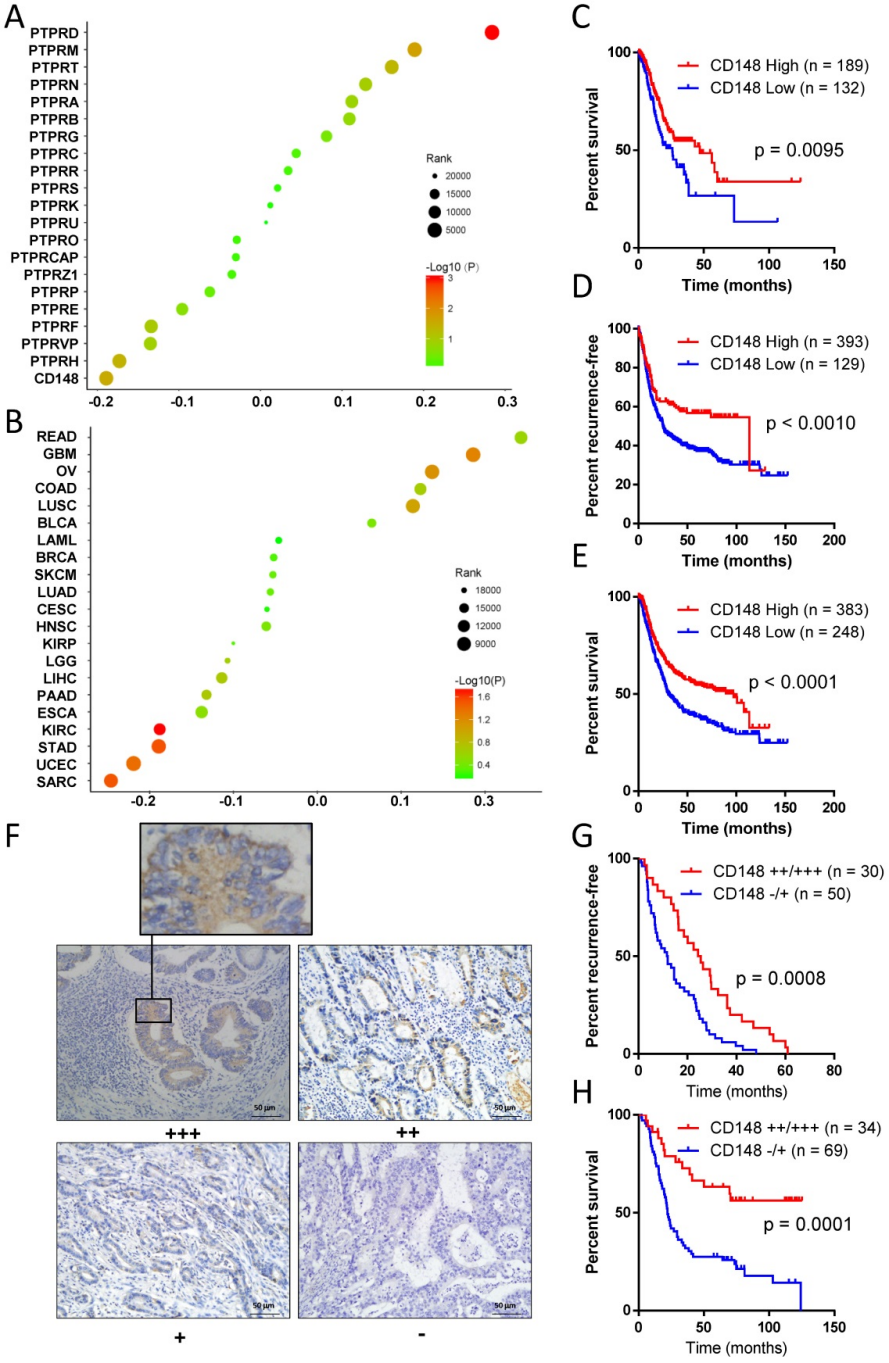
** CD148 levels correlate with prognosis in gastric cancer patients.** (A) Correlations of PTPR members with prognosis in gastric cancer patients. Cox coefficients (x-axis) were obtained by regression of expression levels of all PTPR members (y-axis) and survival time. (B) Pan-cancer analysis of prognostic values of CD148. In A and B, colors of dots represent P values, and sizes of dots represent ranks of Cox coefficients in all genes. (C) Kaplan-Meier plot of survival data in patients with CD148 high *vs.* low expression. Data were derived from TCGA databases. Kaplan-Meier plots of (D) recurrence-free survival and (E) overall survival with CD148 high *vs.* low expression. Data were interrogated from the GEO datasets GSE14210, GSE15459, GSE22377, GSE29272, GSE38749, GSE51105, and GSE62254. (F) Representative immunohistochemistry staining of CD148 in gastric cancer tissues (200×). One representative sample of staining intensities -, +, ++, and +++ are shown. Kaplan-Meier plots of (G) recurrence-free survival and (H) overall survival in patients with CD148 ++/+++* vs.* -/+ staining. Patients were divided into two groups according to the immunohistochemistry staining score. Log-rank tests were used to compare the differences between the two groups in C-E and G-H.

To explore key PTPRs that affect gastric cancer prognosis, we performed Cox regression analyses for all PTPR members with patients' survival data in TCGA databases. CD148 had the most prominently negative correlation with the prognosis of gastric cancer (Cox coefficient = -0.189, *P* = 0.029), indicating that CD148 may inhibit gastric cancer progression (Figure 1A). Meanwhile, PTP receptor type D (PTPRD) displayed the most significant positive correlation with the prognosis of gastric cancer (Cox coefficient = 0.284, *P* = 0.0001; Figure 1A). After a review of the literature, we found that the role of PTPRD in gastric cancer had been reported and, thus, we focused on CD148. Pan-cancer analyses showed that CD148 expression negatively correlated with prognosis of multiple malignancies besides gastric cancer, including sarcoma, endometrial cancer, and renal clear cell carcinoma, with statistical significance (*P* < 0.05; Figure 1B). The survival curve showed that patients with high CD148 expression had a better prognosis (*P* = 0.0095, HR = 0.6197, 95% CL = 0.4317-0.8898; Figure 1C). The median overall survival of CD148-high patients is much longer than that of the CD148-low patients (46.9 *vs.* 25.9 months, Figure 1C).

The online tool Kaplan-Meier Plotter (http://kmplot.com) was used to verify the former discovery and reached the same conclusion. Patients with high CD148 expression showed a longer recurrence-free survival (*P* = 0.0010, HR = 0.6209, 95% CI = 0.5022 - 0.8390; Figure 1D) and overall survival (*P* < 0.0001, HR = 0. 6365, 95% CI = 0.5075 - 0.7821; Figure 1E). The median recurrence-free survival and overall survival in CD148-high patients were up to 97.0 and 113.2 months, whereas those of CD148-low patients were only 24.9 and 30.4 months, respectively.

In addition, we performed immunohistochemistry staining for CD148 expression in gastric cancer samples of 109 patients. CD148 was mainly distributed in the cytoplasm and membrane of tumor cells (Figure 1F). CD148 expression was classified into four grades according to staining intensity: negative (-), weak positive (+), positive (++), and strong positive (+++). In the univariate survival analysis curve, patients with strong CD148 (++ or +++) staining showed a statistically longer recurrence-free survival (*P* = 0.0008, HR = 0.4862, 95% CI = 0.2904-0.7011) and overall survival (*P* = 0.0001, HR = 0.3377, 95% CI = 0.2416-0.6234) than patients with weak CD148 staining (- or +; Figure 1G and H).

### Reduced CD148 expression indicates adverse pathological features of gastric cancer

To further determine the clinical relevance of CD148, we analyzed the correlation between CD148 expression and pathological characteristics of 109 patients (Table 1). The median age of the patients was 57 years, and 76.15% of them were males. The 1-, 3-, and 5-year survival rates were 83.50%, 43.69%, and 34.95%, respectively. According to the American Joint Committee on Cancer (AJCC)-Cancer Staging Manual (Version 8), we found 11 patients in stage I (10.09%), 28 in stage II (25.69%), 48 in stage III (44.04%), and 22 in stage IV (20.18%).

According to staining intensity, 33, 40, 20, and 16 cases expressed CD148 with scores (-), (+), (++), and (+++). CD148 expression correlated significantly with AJCC staging (*P* < 0.0001), lymph node metastasis (*P* < 0.0001), distant metastasis (*P* = 0.0421), vascular invasion (*P* = 0.0270), and differentiation (*P* = 0.0005; Table 1). Instead, no correlation was found between CD148 expression and gender, age, or tumor size.

### CD148 impedes gastric cancer cell proliferation

**Figure 2:**
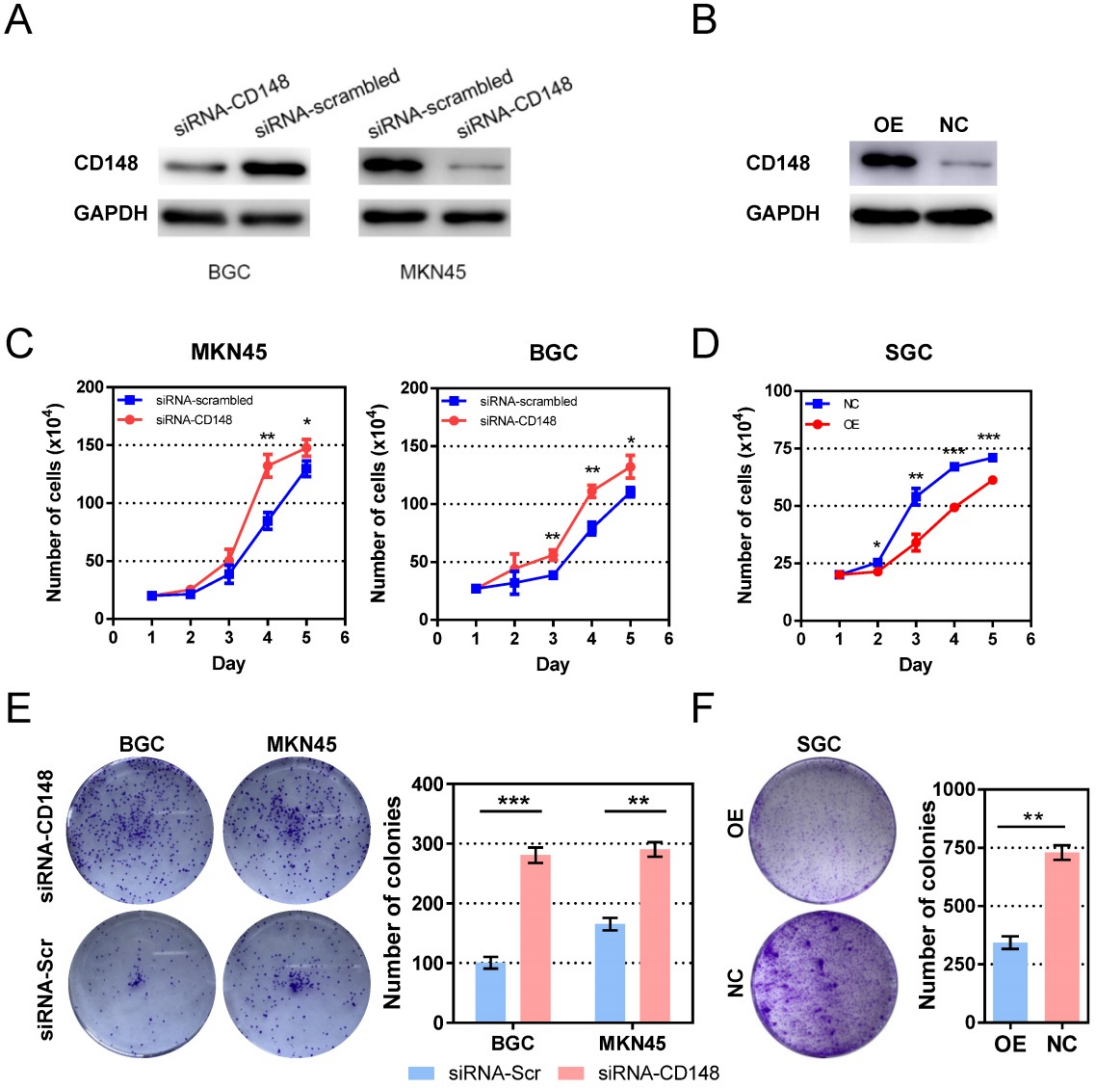
** CD148 impedes gastric cancer cell proliferation *in vitro*.** (A) CD148 expression in BGC and MKN45 cells transfected with CD148 siRNA and scrambled siRNA. (B) CD148 expression in SGC cells transfected with CD148 overexpression plasmid and empty vectors. (C) Proliferation curves of CD148-reducing cell models and controls. (D) Proliferation curves of the CD148-overexpressing cell model and control. Colony-formation assays of (E) CD148-reducing and (F) CD148-overexpressing cell models and controls. Representative plates are shown on the left, and quantification is on the right. Each panel represents at least three independent experiments. Error bars indicate the standard errors of the means. *P < 0.05; **P < 0.01; ***P < 0.001; OE, overexpression; CTL, control; siRNA-Scr, siRNA-scrambled.

To investigate the role of CD148 in tumor growth, we established cell models with CD148 downregulation based on BGC and MKN45 cell lines by siRNA (Figure 2A). The CD148 overexpression model was established based on SGC cell lines by plasmid transfection (Figure 2B). As expected, CD148 downregulation stimulated cell proliferation in both cell lines, and statistical differences rose from day 4 and 3 (*P* < 0.05; Figure 2C). CD148 overexpression significantly impeded cell proliferation (*P* < 0.05 from day 2; Figure 2D).

We also assessed the ability of cell anchorage-independent growth with a colony-formation assay. The BGC and MKN45 obtained a superior ability in colony formation when their CD148 was downregulated (*P* = 0.0004 and 0.0015; Figure 2E). Again, CD148 overexpression decreased colony-forming efficiencies of SGC cells (*P* = 0008; Figure 2F).

### CD148 inhibits gastric cancer cell motility and invasiveness

**Figure 3:**
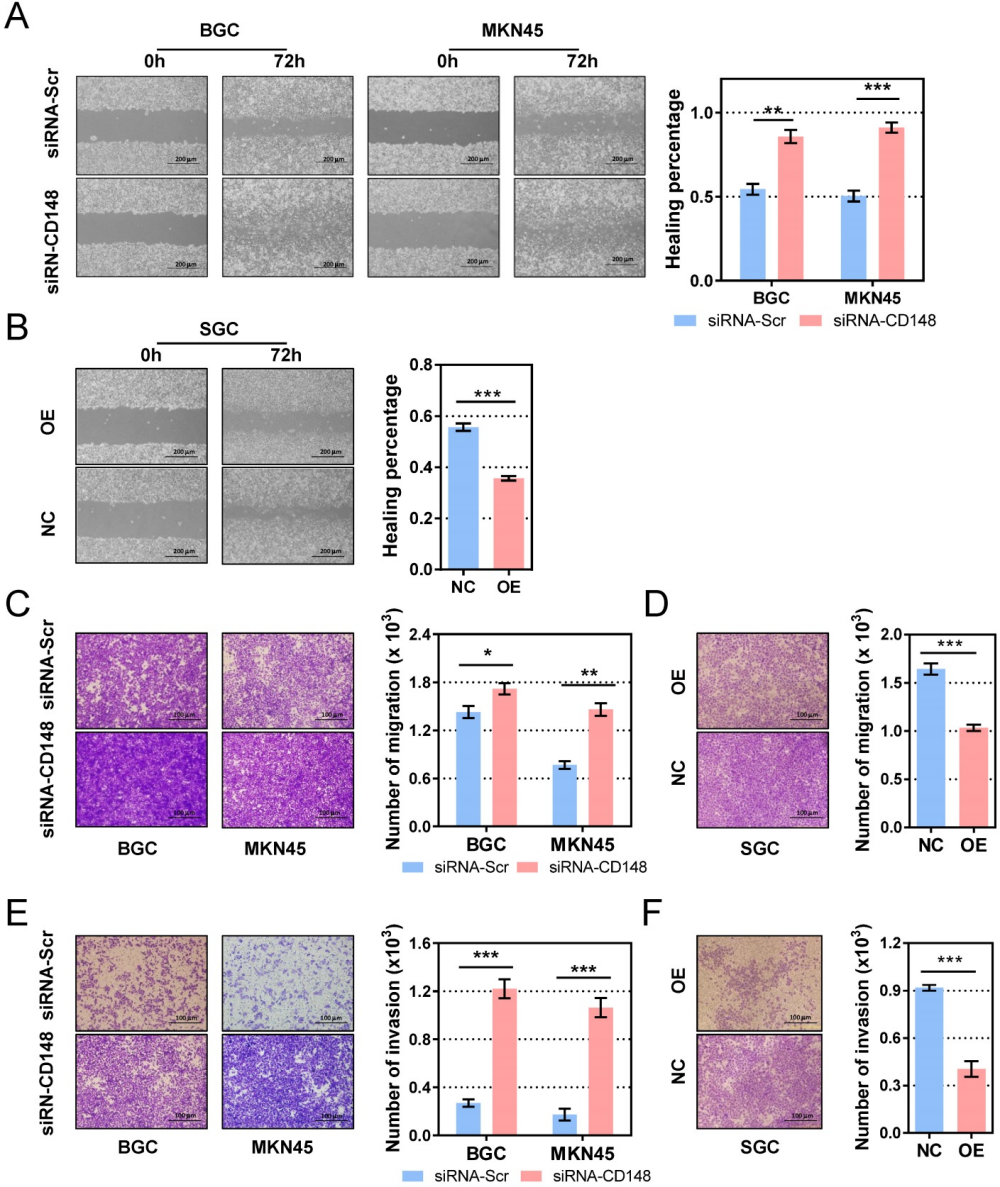
** CD148 inhibits migration and invasion of gastric cancer cells *in vitro*.** Wound-healing assays in (A) CD148-reducing and (B) CD148-overexpressing cell models and controls. Cell migration assays in (C) CD148-reducing and (D) CD148-overexpressing cell models and controls. Cell invasion assays in (E) CD148-reducing and (F) CD148-overexpressing cell models and controls. Representative images (100× in A-B and 200x in C-F) are shown on the left, and quantification on the right. Each panel represents at least three independent experiments. Error bars indicate the standard errors of the means. *P < 0.05; **P < 0.01; ***P < 0.001; OE, overexpression; CTL, control; siRNA-Scr, siRNA-scrambled.

Migration and invasion are essential steps for tumor metastasis, so we examined the role of CD148 on gastric cancer cell motility. In the cell-scratch assay, CD148 downregulation by siRNA significantly augmented the migration distance in both BGC cells (*P* = 0.0034) and MKN45 cells (*P* = 0.0085; Figure 3A). In contrast, CD148 upregulation remarkably reduced migratory ability of SGC cells (*P* = 0.0003; Figure 3B). In Transwell assays, CD148-downregulated cells showed superior migration (*P* = 0.0489 in BGC and 0.0016 in MKN45; Figure 3C) and invasion (*P* = 0.0004 in BGC and 0.0097 in MKN45; Figure 3E) compared to control cells. Accordingly, CD148 overexpression slowed migration (*P* = 0.0008; Figure 3D) and invasion (*P* = 0.0006; Figure 3F) in SGC cells.

### CD148 suppresses gastric cancer growth *in vivo*

**Figure 4:**
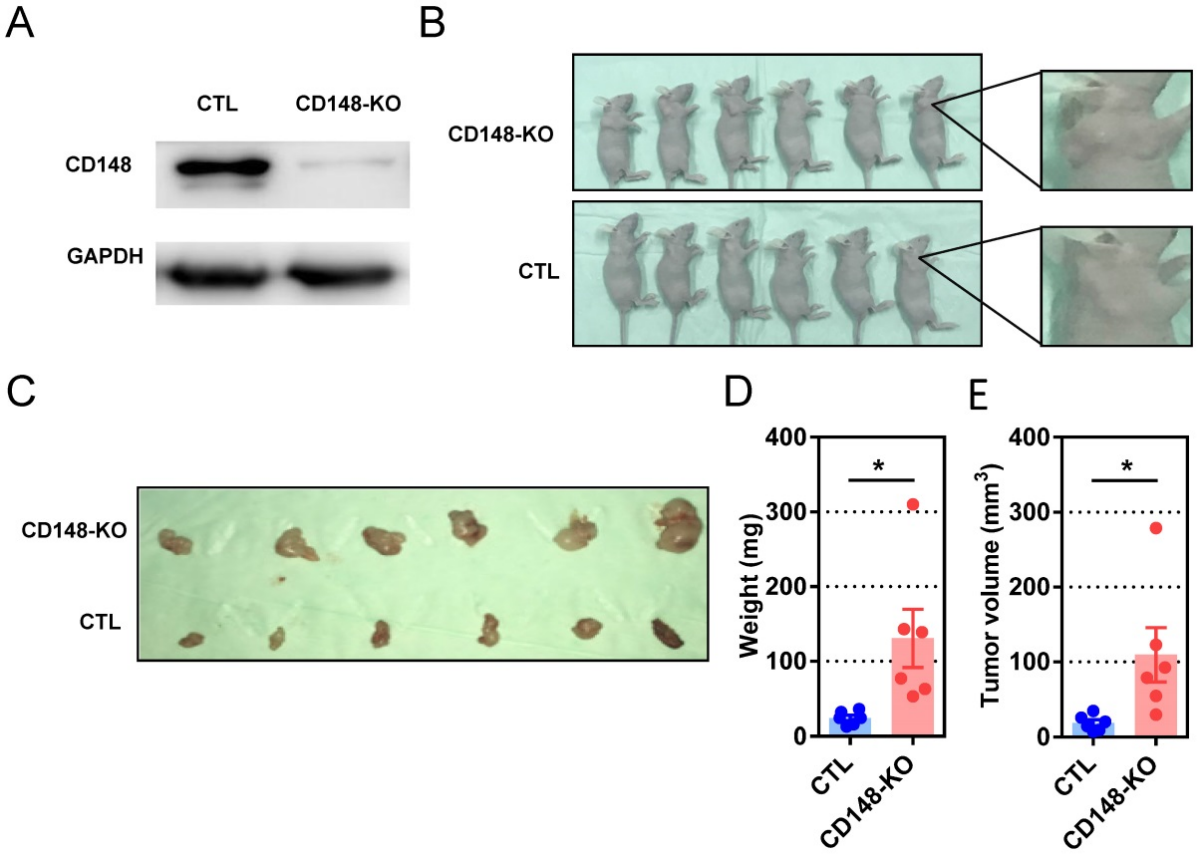
** CD148 suppresses gastric cancer tumor growth *in vivo*.** (A) CD148 expression levels in cells with CD148 CRISPER/cas9 or control vector. (B) Tumor-harboring mice 4 weeks post-implantation of CD148-knockout cells (n = 6) and control cells (n = 6). (C) Xenograft tumors of CD148-knockout cells and control cells 4 weeks post-injection. Tumor xenograft (D) weights and (E) volumes. Each dot represents one sample. Error bars indicate the standard errors of the means. *P < 0.05; KO, knockout; CTL, control.

We next evaluated the influence of CD148 on carcinogenesis and progression of subcutaneous xenografts in nude mice. Mice were subcutaneously inoculated with cells genetically edited with CRISPR/cas9 targeting CD148 or vector control (Figure 4A), then analyzed at week 4 (Figure 4B and C). Although palpable masses were identified on all mice with or without CD148, tumors in the CD148-knockout group were remarkably heavier (*P* = 0.0217; Figure 4D) and larger (*P* = 0.0321; Figure 4E). These data suggest that CD148 confers an onco-suppressing role *in vivo*.

### CD148 dephosphorylates EGFR and suppresses downstream MEK/ERK and PI3K/AKT pathways

**Figure 5:**
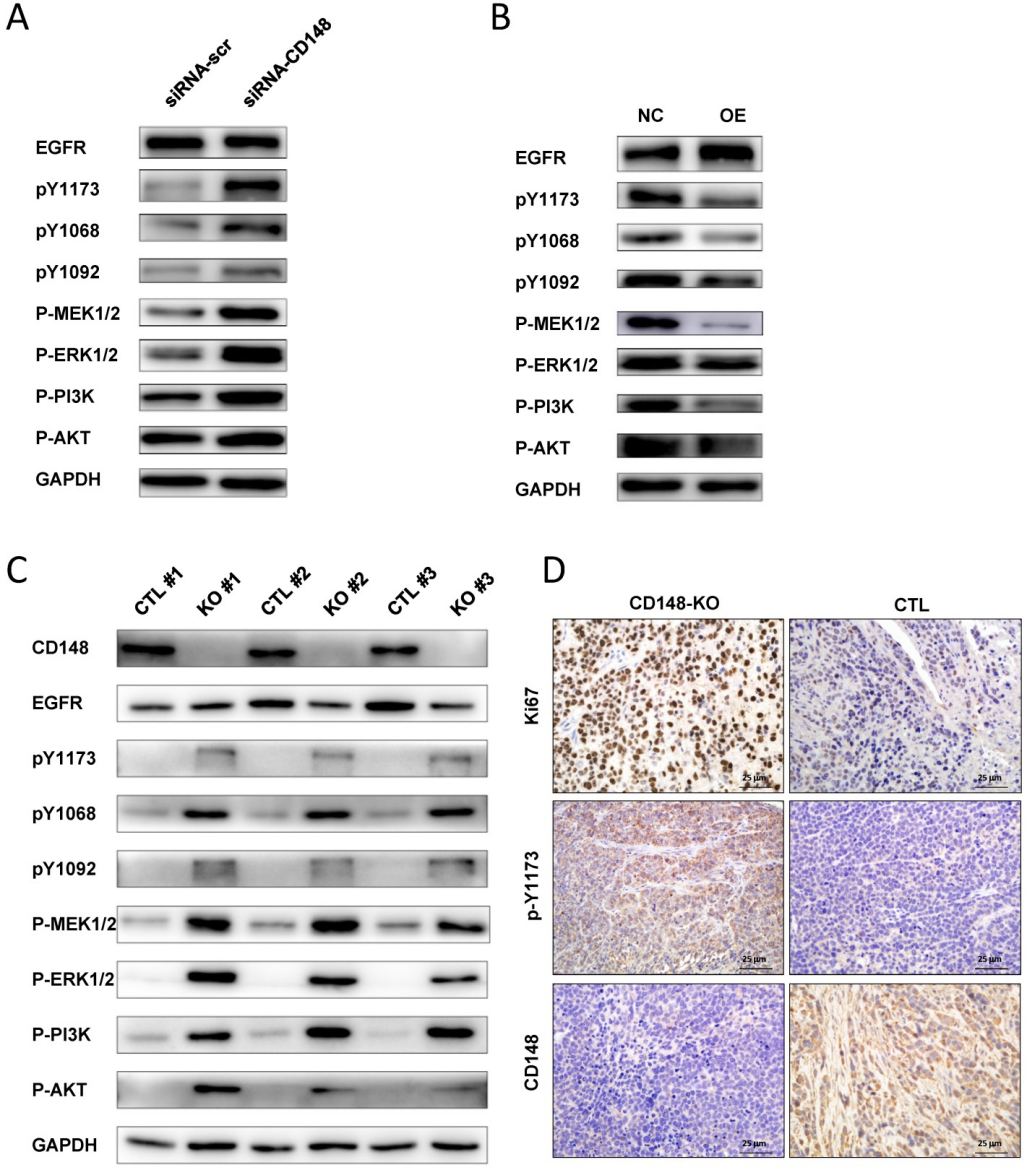
** CD148 regulates EGFR dephosphorylation and downstream pathways in gastric cancer.** Western blot analysis with indicated antibodies in (A) CD148-reducing cells and (B) CD148-overexpressing cells. (C) Western blot analysis with the indicated antibodies in xenograft tumors (n = 3 for mice injected with CD148-knockout cells, n = 3 for mice injected with control cells). (D) Immunohistochemistry staining (400×) of xenograft tumors with indicated antibodies. CTL, control; OE, overexpression; KO, knockout.

EGFR is overexpressed in more than 30% of gastric cancers and plays essential roles in gastric cancer progression 27, 28. It is a target of CD148, but the regulation of EGFR by CD148 has not been studied in certain cancers 16. Therefore, we examined whether CD148 affected EGFR and downstream signaling pathways in gastric cancer. In CD148-knocked-down cells, the level of total EGFR changed little, but levels of phosphorylated EGFR at Y1173, Y1068, and Y1092 sites were all significantly increased (Figure 5A). Accordingly, downstream players of EGFR, including PI3K, AKT, MEK1/2, and ERK1/2, were all profoundly phosphorylated in these cells (Figure 5A). In contrast, levels of phosphorylated Y1173, Y1068, and Y1092, as well as phosphorylated MEK1/2 and PI3K, were remarkably depressed in CD148-overexpressing cells (Figure 5B). The levels of phosphorylated ERK1/2 and AKT were also slightly downregulated in these cells (Figure 5B).

In addition, we verified the EGFR-dephosphorylating role of CD148 in animals. The CD148-downregulated tumors displayed slightly reduced EGFR and dramatically high levels of pY1173, pY1068, and pY1092 (Figure 5C). PI3K, AKT, MEK1/2, and ERK1/2 were also significantly phosphorylated in these tumors (Figure 5C). Phosphorylation of EGFR (Y1173) by CD148* in vivo* was also confirmed by IHC staining (Figure 5D). In addition, Ki67 expression was significantly upregulated by CD148 knockout (Figure 5D). Taken together, these data suggest that CD148 regulates EGFR phosphorylation at multiple tyrosine residues and inhibits activation of downstream PI3K/AKT and MEK/ERK pathways.

### DNA methylation in the 3' UTR region of *PTPRJ* gene is closely associated with CD148 expression

**Figure 6:**
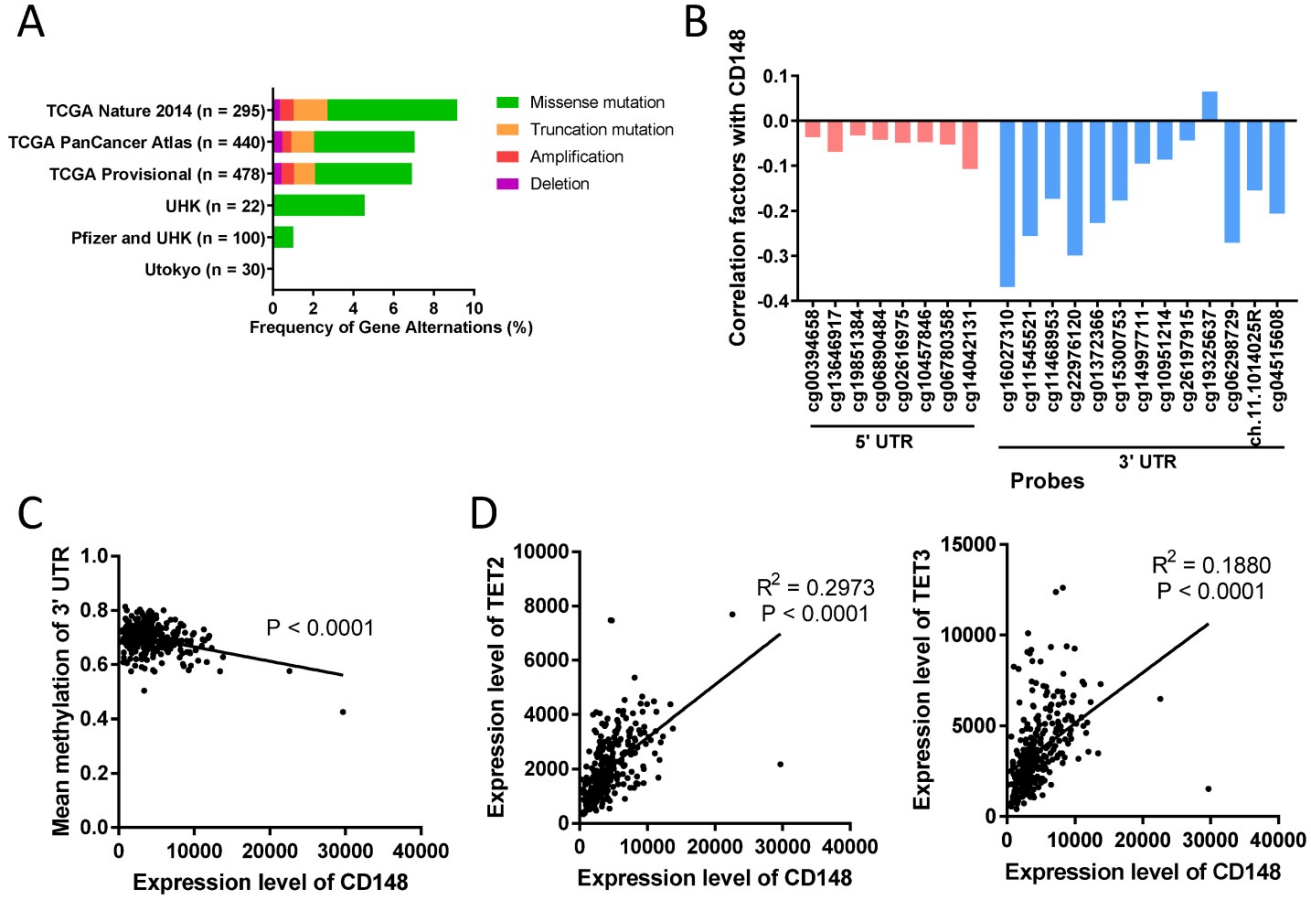
** DNA methylation in the 3' UTR region of *PTPRJ* gene associates with CD148 expression.** (A) Genetic changes in gastric cancer cohorts from online databases. Original data were extracted from cBioPortal. (B) Correlation of CD148 expression and DNA methylation levels. Values indicate Pearson correlation coefficients (y-axis). Red bars indicate 5' UTR probes, and blue bars indicate 3' UTR probes. (C) Correlation between mean methylation levels of 3' UTR probes (y-axis) and CD148 expression (x-axis) in TCGA gastric cancer cohorts. (D) Correlation between TET2/TET3 expression (y-axis) and CD148 expression (x-axis) in TCGA gastric cancer cohorts.

In some tumors, including colorectal cancer, thyroid cancer, and meningioma, CD148 expression levels were altered by gene deletion 29, 30. To determine what alters CD148 expression in gastric cancer, we first analyzed the genetic changes in 1365 gastric cancer patients using the cBioPortal database (http://www.cbioportal.org). We found that *PTPRJ* gene deletions only accounted for less than 0.45% in these patients (Figure 6A). In addition, gene mutations, including missense mutations and truncated mutations, only accounted for 0.00% to 8.13% of total patients (Figure 6A).

DNA methylation is another major cause of gene expression dysregulation. Therefore, we analyzed the DNA methylation status of the *PTPRJ* gene from TCGA cohorts using the online tool Wanderer (http://maplab.imppc.org/wanderer). Methylation on most loci around *PTPRJ* genes negatively correlated with CD148 expression (Figure 6B). In particular, methylation in the 3' UTR region negatively correlated with CD148 expression (R = 0.1779, *P* < 0.0001; Figure 6C), while that in the 5' UTR did not (*P* = 0.2403).

To find the upstream regulators of this site-specific methylation, we analyzed the correlation of multiple methylases (DNMT1, DNMT3A, DNMT3B, and DNMT3L) and demethylases (TET1, TET2, TET3, and TDG) with CD148 expression patterns. TET2 and TET3 were highly correlated with CD148 expression (R = 0.4204, *P* < 0.0001 and R = 0.2658, *P* < 0.0001; Figure 6D). Taken together, we found a possible site-specific methylation for CD148 expression in gastric cancer, with potential regulators TET2 and TET3.

## Discussion

Increasing evidence has shown that PTPRs play an important role in tumorigenesis and progression. CD148 is a member of the PTPR family encoded by the *PTPRJ* gene. Though CD148 was found dysregulated in several tumors, its role in cancer remains controversial. For instance, CD148 inhibits cancer growth and metastasis in colon cancer 17, breast cancer 18, and thyroid cancer 19, but promotes metastasis in breast cancer 21 and glioma cells 22. Moreover, the biological significance of CD148 in gastric cancer has not yet been reported. In this study, we found that CD148 was closely associated with the prognosis of gastric cancer through analyses of clinical databases. Immunohistochemistry showed that high CD148 expression corresponded to long recurrence-free and overall survival in gastric cancer patients, mature differentiation, early AJCC stage, few lymph nodes or distant metastases, and few vascular invasions. Further, CD148 inhibited proliferation, migration, invasion, and colony formation of gastric cancer cells *in vitro*, and also hindered tumor progression *in vivo*. Thus, we have reached a definite conclusion that CD148 plays an onco-suppressing role in gastric cancer. Based on these findings, we speculate that CD148 has potential as a prognostic biomarker and as a therapeutic target in gastric cancer. In addition, we noted a contradiction that the SGC cells expressed low endogenous CD148 but proliferated slowly *in vitro*, compared to the BGC and MKN45 cells. This might be due to other factors, such as genetic, epigenetic, or proteic factors that influenced tumor proliferation.

The inconsistent effects of CD148 in cancers are probably due to the diversity of its substrates in different cancers. CD148 may dephosphorylate ERK1/2 13, 19, FDGFR 31, VEGFR 32, Src 15, 21, 33, and FYN 22 in cell lines or animal models. Dephosphorylation of ERK1/2, PDGFR, and VEGFR by CD148 attenuates downstream pathways to inhibit tumor progression 13, 19, 31, 32, while dephosphorylation of Src and FYN activates them and promotes tumor progression 15, 21, 22, 34. The reason for this contradiction is that phosphorylation of certain loci on Src and FYN may inhibit downstream signal transduction, which explains why CD148 exhibits a tumor-promoting effect in some tumors.

EGFR is an essential player in gastric cancer, and overexpression of EGFR was detected in more than 30% of patients 27, 28. Activation of EGFR requires phosphorylation at tyrosine loci (such as Y1173), homo-dimerization, and internalization into the cytoplasm 35. Recent studies have reported that EGFR is also a dephosphorylation substrate for CD148 in HEK293 cells; CD148 counters phosphorylation at multiple sites in EGFR, formation of homodimers, and subsequent endocytosis and degradation 16. However, there is no report that CD148 can inhibit malignant phenotypes of cancer cells by EGFR dephosphorylation. Our study revealed that CD148 exerted onco-suppressing function by dephosphorylating EGFR on Y1173, Y1068, and Y1092 and inhibiting the MEK/ERK and PI3K/AKT pathways in gastric cancer. Previous studies also found that CD148 directly dephosphorylated ERK1/2 to inhibit the RAS signaling pathway 13. Whether CD148 dephosphorylates ERK1/2 directly or through EGFR-deactivation in gastric cancer cells will be clarified in future work.

Several mechanisms regulate CD148 expression or function, but mechanistic studies in gastric cancer have not yet been reported. Loss of heterozygosity at the *PTPRJ* locus is frequently found in certain tumors, including lung cancer, breast cancer, and colorectal cancer 29, 30. Missense polymorphism is also considered a mechanism to affect molecular functions of CD148 as well as cancer susceptibility 34. In addition, miRNAs, such as miRNA-328 or miRNA-155, regulate expression of CD148 in colorectal cancer 36. In comparison, only a few of the gastric cancer samples showed deletions (5/1365), missense mutations (66/1365), or truncation mutations (15/1365), indicating a low incidence of these mechanisms in gastric cancer. Instead, we found a high level of DNA methylation in the 3' UTR region of *PTPRJ* gene and a close correlation between methylation level and CD148 expression. Therefore, DNA methylation may be a key regulator of CD148 expression in gastric cancer.

A couple of limitations exist in this study. First, the DNA methylation study of the *PTPRJ* gene was only performed *in silico*, and thus the conclusion awaits validation in future *in vitro* studies. Second, we did not investigate the TSP-1, the serum ligand of CD148 and a deactivator of EGFR 37. This molecule may exert its onco-suppressing role by activating CD148, and its potential as a therapeutic target for gastric cancer should be evaluated. Third, although revealed in previous studies 16, direct evidence of how CD148 dephosphorylate the Y1173, Y1068, and Y1092 on EGFR was lacked in gastric cancers. Fourth, the role of CD148 in metastasis was not confirmed in *in vivo* models. These remain to be explored in future studies.

## Conclusions

In summary, CD148 may serve as a prognostic factor in gastric cancer, and its downregulation might be a molecular abnormality linked to oncogenesis and metastasis of gastric cancer through EGFR phosphorylation and subsequent downstream signaling activation. Therefore, CD148 and its related proteins could be used as a potential predictive marker and candidate therapeutic target to improve the prognosis of gastric cancer patients.

## Figures and Tables

**Table 1:** CD148 expression correlates with clinicopathological features in human gastric cancer tissue

Clinicopathological features	Number ^†^	CD148 expression^ †^		P value ^‡^
		Low	High	
Gender				0.4482
Male	83 (75.5%)	54 (65.1%)	29 (34.9%)	
Female	27 (24.5%)	19 (73.1%)	7 (26.9%)	
Age				0.8403
≥ 65	23 (21.1%)	15 (65.2%)	8 (34.8%)	
< 65	86 (78.9%)	58 (67.4%)	28 (32.6%)	
**Stage**				**< 0.0001**
I+II	39 (35.8%)	14 (35.9%)	25 (64.1%)	
III+IV	70 (64.2%)	59 (84.3%)	11 (15.7%)	
**T stage**				0.2597
T1 or T2	18 (15.5%)	10 (55.6%)	8 (44.4%)	
T3 or T4	91 (83.5%)	63 (69.2%)	28 (30.8%)	
**Lymph node metastasis**				**< 0.0001**
Yes	78 (71.6%)	63 (80.8%)	15 (19.2%)	
No	31 (28.4%)	10 (32.3%)	21 (67.7%)	
**Metastasis**				**0.0421**
M0	88 (80.7%)	55 (62.5%)	33 (37.5%)	
M1	21 (19.3%)	18 (85.7)	3 (14.3%)	
**Differentiation**				**0.0005**
Poorly	59 (54.1%)	48 (81.4%)	11 (18.6%)	
Well	50 (45.9%)	25 (50.0%)	25 (50.0%)	
**Microvascular invasion**				**0.0270**
Present	18 (27.3%)	16 (88.9%)	2 (11.1%)	
Absent	48 (72.7%)	29 (60.4%)	19 (39.6%)	

†Data are presented as numbers (proportions). ‡P values were calculated by Chi-square test or Fisher's exact test. Statistically significant correlations are highlighted in bold.

## Abbreviations

DEP-1: density-enhanced phosphatase 1
PTK: protein tyrosine kinase
PTP: protein tyrosine phosphatases
PTPR: receptor-type protein tyrosine phosphatases
PTPRD: protein tyrosine phosphatase, receptor-type D
PTPRJ: protein tyrosine phosphatases, receptor-type J
